# Sirtuins—Novel Regulators of Epigenetic Alterations in Airway Inflammation

**DOI:** 10.3389/fgene.2022.862577

**Published:** 2022-05-10

**Authors:** Shunyu Wu, Huanhai Liu

**Affiliations:** Department of Otolaryngological, the Second Affiliated Hospital of the Naval Military Medical University (Shanghai Changzheng Hospital), Shanghai, China

**Keywords:** sirtuins, epigenetic alterations, histone modification, airway inflammation, modulators

## Abstract

Histone modification is an important epigenetic alteration, and histone deacetylases are involved in the occurrence and development of various respiratory diseases. Sirtuins (SIRTs) have been demonstrated to play an important role in the formation and progression of chronic inflammatory diseases of the respiratory tract. SIRTs participate in the regulation of oxidative stress and inflammation and are related to cell structure and cellular localization. This paper summarizes the roles and mechanisms of SIRTs in airway inflammation and describes the latest research on SIRT modulators, aiming to provide a theoretical basis for the study of potential epigenetic alteration-inducing drug targets.

## 1 Introduction

Chronic inflammation is characterized by the presence of several pro-inflammatory mediators, primarily produced by activated macrophages, such as interleukin 1β (IL-1β), tumor necrosis factor-α (TNF-α), and interleukin 6 (IL-6) which persist in inflammation (Kany et al., 2019). Inflammatory signaling is mediated by the activation of enzymes and adhesion molecules as well as central regulators such as nuclear factor KB (NF-KB) and other transcription factors, and the immune system and associated cells also play a coordinating role (Bondia-Pons et al., 2012). In healthy individuals, inflammatory cells are relatively quiescent and effectively protect the body against infectious agents. When infectious organisms invade the body, inflammatory cells rapidly proliferate and differentiate into functional inflammatory cells that perform several functions, exhibiting altered gene expression profiles and secreting cytokines (Alves-Fernandes and Jasiulionis, 2019). Identifying the factors associated with the onset and progression of inflammation is critical for understanding inflammation-related diseases and determining therapeutic targets. Emerging evidence suggests that epigenetic processes may contribute to the pathophysiology of inflammatory processes that occur after environmental stimulation and play an important role in the transcription of inflammatory genes (Bayarsaihan, 2011).

**FIGURE 1:**
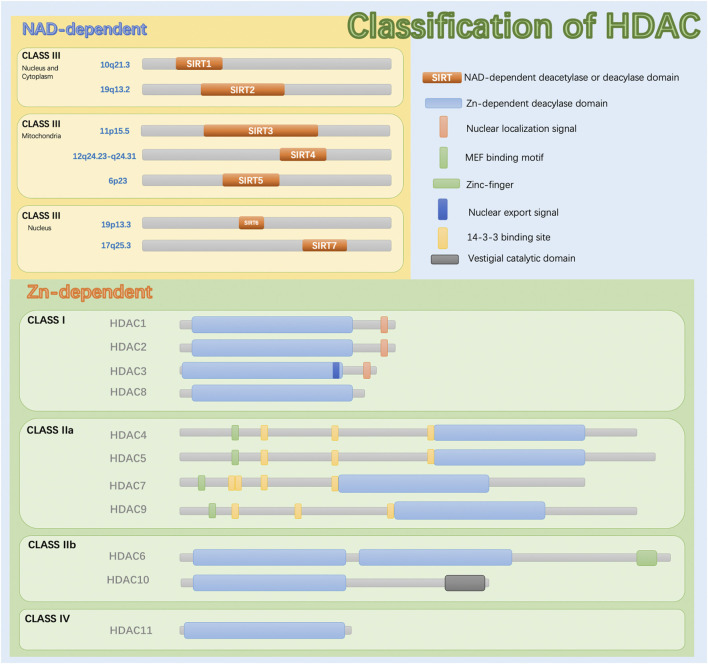
The classification and molecular structure of HDAC.

Epigenetic modifications are heritable changes that do not involve changes in gene expression levels or alterations in DNA sequences. Abnormal epigenetic modifications can cause structural and functional changes in the body and cause disease (Loscalzo and Handy, 2014). Epigenetic modifications are mainly regulated by DNA methylation, histone acetylation or methylation, non-coding RNA (ncRNA) modification, etc., and each regulatory process can act alone or interact with others (Ramos-Lopez et al., 2021). In particular, histones, important components of nucleosomes in chromatin, can be acetylated or methylated. Histone deacetylases (HDACs) are a class of enzymes responsible for the deacetylation (removal of acetyl groups) of lysine residues in the tail of histones (Figure 1). Histone deacetylation strengthens the interaction between histones and DNA, prompting DNA to wrap more tightly around the histones. This leads to a more compact chromatin structure and results in gene silencing. HDACs can also modify several non-histones and interact with various transcription factors to further regulate gene transcription. Specific inhibition of these enzymes with HDAC inhibitors (HDACis) can counteract their effects and alter gene expression by promoting active transcription (Raghuraman et al., 2016). As mentioned above, HDACs are involved in the occurrence and development of various respiratory diseases, including bronchial asthma (BA), chronic sinusitis (CRS), chronic obstructive pulmonary disease (COPD), acute lung injury (ALI), and acute respiratory distress syndrome (ARDS), which will be described in detail below.

Due to the complexity of inflammatory cells, the pathogenesis of respiratory system diseases is multifaceted. Some researchers believe that those blood-based molecular parameters and other metabolites, such as DNA methylation markers, cell-free DNA mutation markers, and microRNAs, are attractive future biomarkers for diagnosing inflammatory diseases (Tsai et al., 2019). Thus, the roles of specific enzymes, especially sirtuins (SIRTs), are particularly interesting. SIRTs can modify proteins through deacetylation and play a role in bronchial asthma. Thus, these enzymes are promising diagnostic and therapeutic markers. Many recent studies have shown that SIRTs are involved in respiratory diseases such as asthma, COPD, and rhinitis (Matucci et al., 2021). In addition, age-related diseases can arise when physiological anti-inflammatory and antioxidant mechanisms fail to protect the body from damage caused by chronic low-grade inflammation and increased levels of reactive oxygen species (ROS). Chronic low-grade inflammation (hereafter, inflammation) associated with aging, for example, plays a major role in the pathogenesis of COPD and stems from an imbalance in inflammatory and anti-inflammatory networks (Franceschi et al., 2017). This difference increases with age, leading to increased susceptibility to disease. COPD-associated inflammation is characterized by an increase in the number of immune cells (e.g., alveolar macrophages, neutrophils, and T lymphocytes) that secrete cytokines, chemokines, growth factors, and lipid mediators, thereby perpetuating the inflammatory process and the destruction of lung parenchyma (Barnes, 2016).

Respiratory diseases are highly heterogeneous, with complex etiologies and unclear pathogeneses. Even diseases within the same clinical classification exhibit large differences in intrinsic cellular and molecular pathological mechanisms. Despite the dramatic increase in the use of new therapeutics for respiratory diseases, they are infrequently discovered; therefore, few new classes of therapeutics have been developed to regulate or treat airway inflammatory diseases. In summary, methods of reestablishing regulatory mechanisms that attenuate this chronic inflammation are urgently needed (Feldman et al., 2012). However, a relationship between SIRTs and respiratory mucosal cell differentiation and function has not previously been reported. This article discusses the roles and mechanisms of SIRTs in airway inflammation and reviews the SIRT modulators developed to date, providing a theoretical basis for the study of potential drug targets directed toward epigenetic alterations.

## 2 SIRTs in the HDAC Family

### 2.1 The Basic Features of SIRTs

Members of the HDAC family contain a highly conserved deacetylase domain and are critical for maintaining the balance of histone lysine acetylation and deacetylation (Peserico and Simone, 2011). The deacetylation of histones leads to chromatin aggregation, which is closely related to transcriptional silencing (Shahbazian and Grunstein, 2007). The most studied type of histone modification is lysine acetylation. For example, neutrophilic airway inflammation is associated with increased histone acetyltransferase (HAT) activity and decreased HDAC activity in peripheral blood mononuclear cells but not with alterations in the gene expression of individual enzymes (Gunawardhana et al., 2014). Similarly, decreases in activity and mRNA expression levels of HDAC1 and HDAC2 have been observed in patients with asthma (Ito et al., 2002).

A SIRT was initially discovered in *Saccharomyces cerevisiae*, silent information regulator 2 (Sir2), and SIRTS were subsequently found in other species, including mammals, in which they play essential roles in epigenetic regulation (Wasti et al., 2021). Eighteen types of human HDACs have been identified and divided into four categories according to functional and genetic criteria (Haberland et al., 2009). HDACs differ in enzymatic function, structure, expression pattern, and subcellular positioning. In addition to their nuclear effects, HDAC isotypes play a role in basic cytoplasmic function by controlling the acetylation activity and status of many cytoplasmic proteins, including transcription factors (Barneda-Zahonero and Parra, 2012). SIRTs are class III HDACs dependent on nicotinamide-adenosine dinucleotide (NAD). They are classified into seven types (SIRT1 to SIRT7) and have been highly conserved throughout evolution. Different SIRTs have similar catalytic domains and have NAD + as a co-substrate. In addition, SIRTs exhibit different substrate affinities and subcellular localizations. SIRTs not only act on histones but also have other cellular targets such as transcription factors and metabolic enzymes (Kosciuk et al., 2019).

**FIGURE 2:**
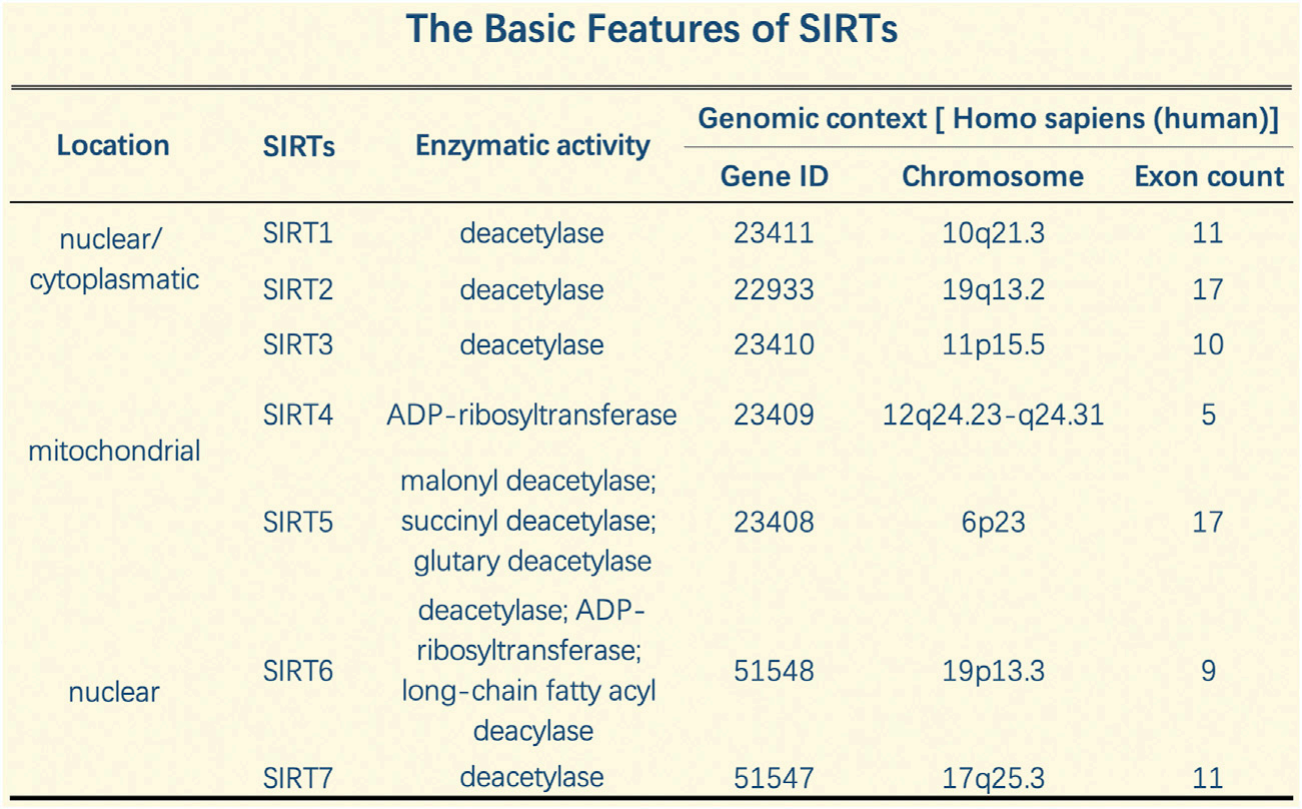
The basic features and genomic context of SIRTs.

In addition, SIRTs exhibit a variety of catalytic activities, and these pleiotropic enzyme activities play important roles in silencing regulatory proteins, maintaining genomic integrity, regulating metabolic homeostasis, and promoting organism longevity (Salekeen et al., 2021). SIRTs share an NAD + -binding catalytic domain and act specifically on different substrates based on the biological processes in which they are involved. SIRTs have different N-terminal and C-terminal domain sequences and lengths, which explain their different localizations and functions. SIRT can catalyze deacetylation and adenosine diphosphate (ADP) ribosylation (Carafa et al., 2012). SIRTs typically perform NAD + -dependent lysine deacetylation; however, recent studies have shown that some SIRTs can remove other acyl groups, such as succinyl, malonyl, glutaryl, and long-chain fatty acyl groups (Tan et al., 2014). While SIRT-dependent deacetylation is the dominant functional role of SIRTs in inflammation according to our current understanding, other properties, such as the role of SIRTs in ADP ribohydrosylation (SIRT4) and the removal of succinyl, malonyl, and glutamyl (SIRT5) from lysine residues, may be important for inflammation (Tao et al., 2015). Specifically, SIRT5 is a highly potent protein lysine desuccinylase and demalonylase *in vitro*. The presence of arginine residues (Arg105) and tyrosine residues (Tyr102) in SIRT5’s acyl bags explains the preference for succinyl and malonyl groups. Several mammalian proteins with succinyl or malonyl lysine modifications were identified by mass spectrometry. Deletion of Sirt5 in mice appears to increase succinylation of carbamoyl phosphytase 1, a known target for SIRT5. Thus, protein lysine succinylation may represent a posttranslational modification that can be reversed by SIRT5 *in vivo* (Du et al., 2011) (Figure 2).

### 2.2 The Activity, Substrates, and Effects of SIRTs Localized in Different Cells

#### 2.2.1 SIRT1 and SIRT2 can Shuttle between the Nucleus and Cytoplasm

**FIGURE 3:**
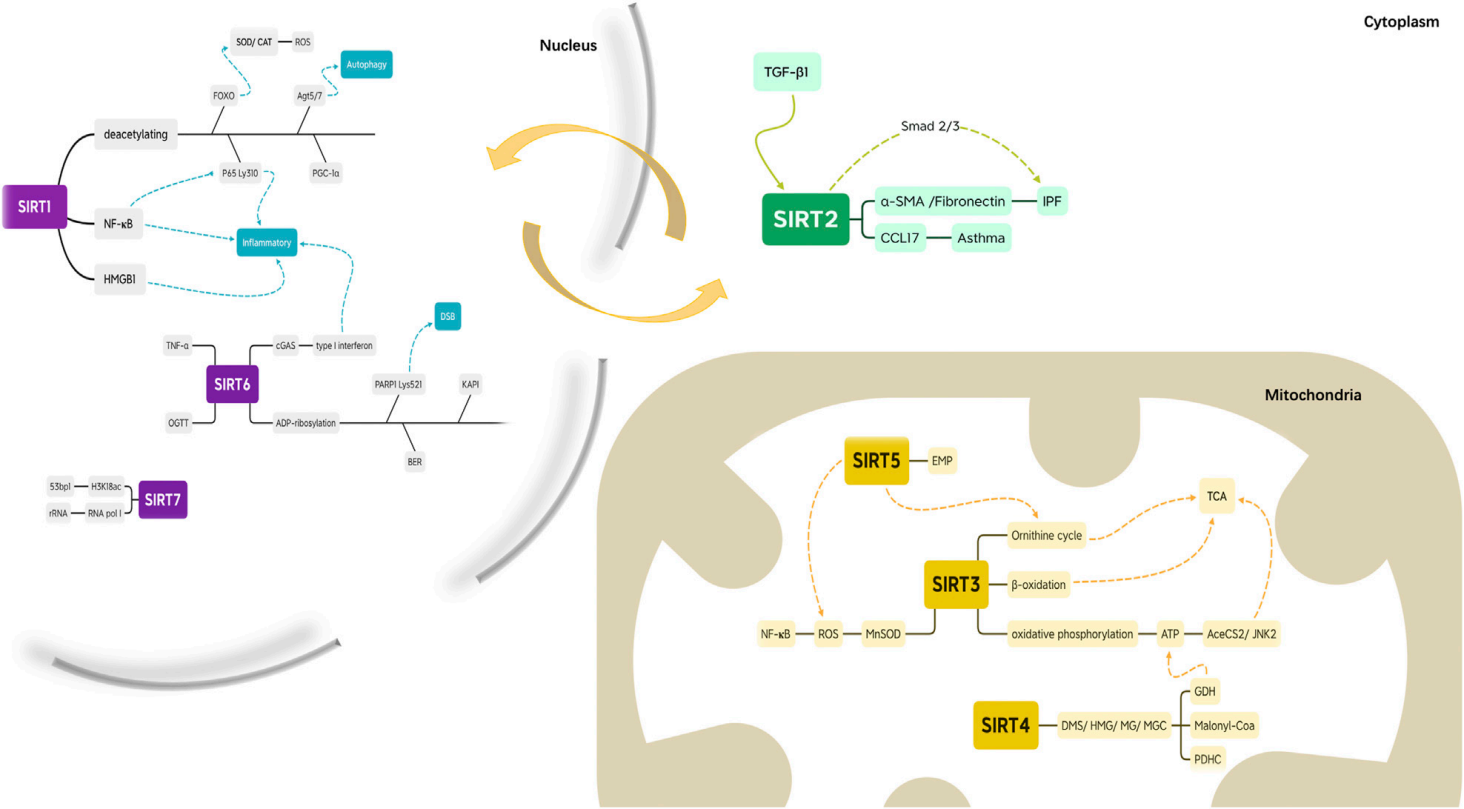
The relationship between cellular locations and enzymatic activity.

SIRTs in the nucleus, mitochondria, and cytosol of undifferentiated cells relocate during cell differentiation (Morris, 2013) (Figure 3). SIRT1 and SIRT2 are localized to the nucleus and cytoplasm. SIRT1, which is mostly located in the nucleus, deacetylates Forkhead Box subfamily O (FOXO) transcription factors to stimulate the expression of manganese superoxide dismutase and catalase, a ROS-deactivating enzyme and deacetylates proliferator-activated receptor coactivator-1a to increase mitochondrial biogenesis and decrease ROS production (Kops et al., 2002; Daitoku et al., 2004; van der Horst et al., 2004; Rodgers et al., 2005). It also weakens the exocytotic effect of the proinflammatory cytokine High Mobility Group Protein 1 (Kim et al., 2016). Moreover, SIRT1 inhibits the activity of the transcription factor NF-KB, suppresses inflammation, and enhances the resolution phase of the inflammatory response by deacetylating the Lys310 residue of the RelA/p65 component of NF-KB (Nopparat et al., 2017). SIRT1 induces autophagy, a mechanism for removing damaged cellular organelles, by deacetylating angiotensinogen 5 (Agt5) and angiotensinogen 7 (Agt7) (Salazar et al., 2020). Overall, SIRT1 is a strong inhibitor of the inflammatory response and reduces oxidative stress (Kauppinen et al., 2013). Hence, an aging-associated decrease in SIRT1 can contribute to chronic inflammation and stress, both of which are hallmarks of inflammation and associated with the accumulation of damaged organelles (including mitochondria), reducing the function of immune cells.

SIRT2 is the only SIRT that is mainly located in the cytoplasm; it colocalizes with microtubules and deacetylates tubulin (North et al., 2003). SIRT2 can move between the nucleus and the cytoplasm via mechanisms that are cell and tissue-specific. SIRT2 is enriched in centrosome and full-intermediate growth spindle fibers (colocalized with γ-tubulin and Aurora A). Unexpectedly, these microtubules appear to be highly acetylated relative to cytoplasmic microtubules (North and Verdin, 2007). Studies have shown that SIRT2 may regulate the acetylation of cellular proteins involved in the cell cycle. Although tubulin is a recognized target for SIRT2, SIRT2 may deacetylate other proteins during the cell cycle (Heltweg et al., 2006). However, it is unclear what role SIRT2 plays in the nucleoplasm during interphase. Future studies should investigate the possibility that interphase nuclear SIRT2 may regulate the expression of gene subsets or the acetylation levels of other nuclear proteins. Recent data indicates that SIRT2 is a debenzoylase both *in vitro* and *in vivo* (He Huang et al., 2018), as SIRT2 removes benzoyl groups from lysine. This type of histone mark can be stimulated by sodium benzoate (SB) via the generation of benzoyl-coenzyme A (CoA). Consistent with its predominant cytosolic location, SIRT2 deacetylates several non-histone proteins (Wang et al., 2017). These results suggest that SIRT2 regulates multiple biological functions, including neurotoxicity, metabolism, mitosis regulation, genome integrity, oxidative stress, and autophagy. One study found that SIRT2 expression was upregulated in transforming growth factor-β1 (TGF-β1)-treated human embryonic lung fibroblasts. A SIRT2 inhibitor or the knockdown of SIRT2 expression by small interfering RNA (siRNA) suppressed the expression of the fibro-genic genes α-SMA and fibronectin in TGF-β1-treated fibroblasts and primary lung fibroblasts derived from patients with idiopathic pulmonary fibrosis (IPF). These data suggest that SIRT2 may be involved in the development of IPF by modulating the Smad2/3 pathway (Gong et al., 2021). Other data suggest that after allergen sensitization and excitation, overexpression or inhibition of SIRT2 modulates the expression of CCL17 and the recruitment of monocytes and macrophages into the airways, so SIRT2 enhances allergic asthma inflammation (Lee et al., 2019).

#### 2.2.2 SIRT3, SIRT4, and SIRT5 are Located in Mitochondria

Mitochondria are organelles critical for regulating cellular metabolism and are central to energy and immune homeostasis during ongoing disease responses (Angajala et al., 2018). As demonstrated by their mitochondrial localization, SIRT3, SIRT4, and SIRT5 are mostly involved in regulating metabolic pathways. These SIRTs regulate basic mitochondrial functions, including ATP production, metabolism, and apoptosis (Kratz et al., 2021).

SIRT3, SIRT4, and SIRT5 are the major mitochondrial deacetylases that regulate stress pathways and energy homeostasis (Ahn et al., 2008), deacetylating several key mitochondrial proteins. SIRT3 promotes the urea cycle, fatty acid β-oxidation, and oxidative phosphorylation and increases the expression and activity of manganese superoxide dismutase, thus reducing ROS production and promoting ROS scavenging (Hallows et al., 2011). Furthermore, SIRT3 reduces oxidative stress, increases stress defenses, and counteracts the development of aging-associated diseases (Jing et al., 2011). This reduction in oxidative stress can, in turn, activate the NF-KB pathway (Chen et al., 2013). SIRT3-knockout mice develop normally but experience changes in mitochondrial protein acetylation and ATP levels (Rardin et al., 2013). After oxidative stress has been induced, SIRT3 coordinates the response to mitochondrial NAD levels to prevent cell death (Yang et al., 2007). SIRT3-driven energetic regulation is further advanced by SIRT3’s role in the tricarboxylic acid (TCA) cycle; it also activates acetyl coenzyme A synthetase 2 (AceCS2) and c-Jun N-terminal kinase 2 (JNK2) (Chen et al., 2014a). Because SIRT3 is a major mitochondrial deacetylase, it is not surprising that SIRT3-knockout mouse embryonic fibroblasts (MEFs) show increased ROS levels (Tao et al., 2010). ROS are removed by manganese-dependent superoxide dismutase (MnSOD), which is deacetylated by SIRT3, affecting MnSOD activity under oxidative stress conditions. Therefore, SIRT3 is essential to combat the mitochondrial oxidative stress as it directly regulates MnSOD (Lombard et al., 2007).

Because of its ADP-ribosyltransferase activity, SIRT4 can be deacetylated (Laurent et al., 2013). Recently, a study showed that SIRT4 catalyzes the removal of lipoyl- and biotinyl-lysine modifications more efficiently than deacetylation (Mathias et al., 2014). In addition, SIRT4 has lysine-deacylase activity for removing 3,3-dimethylsuccinyl (DMS), 3-hydroxy-3-methylpentadienyl (HMG), 3-methylglutaryl (MG) and 3-methylglutamyl (MGc) (Pannek et al., 2017). These enzyme activities enable SIRT4 to regulate the targets of glutamate dehydrogenase (GDH), malonyl-CoA decarboxylase, and the pyruvate dehydrogenase complex (PDHC) directly. Therefore, SIRT4 plays an essential role in the metabolic regulation of lipids, glucose, and proteins (Mathias et al., 2014). For example, SIRT4 interacts with GDH and inhibits its activity, reducing the metabolism of glutamate and limiting ATP production. ADP ribosylation of GDH reduces its enzyme activity, inhibiting the progression of the TCA cycle and regulatory activities such as insulin secretion and cell proliferation (Haigis and Guarente, 2006). SIRT4-knockout mice appear healthy but demonstrate increased mitochondrial GDH activity. More recently, Cristea and others (Mathias et al., 2014) demonstrated that SIRT4, a mitochondrial lipase, plays an even more important role as the guardian of mitochondrial pyruvate entry by directly regulating the mitochondrial PDHC. Thus, it is not surprising that flies lacking SIRT4 exhibit many metabolic defects, including increased sensitivity to hunger, reduced fertility and activity, and an inability to utilize energy reservoirs, especially long-chain fatty acids (Wood et al., 2018). SIRT4 expression may also be associated with improved olfaction, indicating that it has neurorestorative or neuroprotective effects (Marin et al., 2019).

SIRT5 was the first SIRT found to exhibit deacylase activity as opposed to deacetylase activity, functioning as a robust mitochondrial desuccinylase and demalonylase (Du et al., 2011). SIRT5 activates ROS detoxification-related enzymes, elevates mitochondrial integrity and function, and controls the urea cycle and other metabolic pathways (Heinonen et al., 2019). In mice, the genetic ablation of SIRT5 is related to enhanced susceptibility to age-related diseases, including obesity, insulin resistance, fibrosis, neurodegeneration, and cardiac dysfunction, while opposite context-dependent effects of SIRT5 have been reported with respect to tumorigenesis (Carrico et al., 2018). One study showed that SIRT5 regulates cytosol and mitochondrial protein malonylation, with glycolysis as the main target (Nishida et al., 2015). Although SIRT5 can deacylate hundreds of mitochondrial proteins, the specific functions of these modifications remain to be determined.

#### 2.2.3 SIRT6 and SIRT7 are Located in the Nucleus

Enzymatic characterization of SIRT6 revealed three catalytic activities: ADP ribosylation, deacetylation, and fatty acid deacylation (Gertman et al., 2018). The first study to explore the enzymatic activity of SIRT described SIRT6 as a monoadenosine diphosphate ribosyl transferase (Liszt et al., 2005). Interestingly, SIRT6 transfers radiolabels through an intramolecular mechanism, suggesting that SIRT6 may regulate its own activity through ADP ribohydrosylation. However, the physiological significance of auto-ADP-ribosylation remains unclear (Elhanati et al., 2013). SIRT6 promotes resistance to DNA damage, inhibits genomic instability in mouse cells and plays a role in base excision repair (BER) (Mostoslavsky et al., 2006). Recent experiments have revealed a role for SIRT6-mediated ADP ribosylation in DNA repair. An additional ribosylation substrate was also identified—SIRT6 was shown to bind nuclear corepressor protein KAP1, inducing KAP1 mono-ADP ribosylation (Van Meter et al., 2014). More recently, SIRT6 was shown to catalyze ADP ribosylation at the Lys521 residue of poly (ADP-ribose) polymerase 1 (PARP1) to promote double-strand break (DSB) repair under oxidative stress (Van Meter et al., 2016). SIRT6 is expressed in the S phase of the cell cycle but not in the nucleolus during G1, and when overexpressed, SIRT6 inhibits mitosis. Researchers have found that mice overexpressing SIRT6 exhibited improved glucose tolerance, which is a younger hormonal profile, reduced age-related adipose inflammation, and increased physical activity (Kanfi et al., 2012; Roichman et al., 2017; Roichman et al., 2021). These results suggest that SIRT6 is a potential therapeutic target for treating a range of age-related diseases.

Mice deficient in SIRT6 exhibit a severely shortened lifespan, growth retardation, and highly elevated LINE1 (L1) activity. One study showed that SIRT6-deficient cells and tissues accumulate abundant cytoplasmic L1 complementary DNA (cDNA), triggering a strong type I interferon response via activation of cyclic guanosine monophosphate–adenosine monophosphate (GMP–AMP) synthase (cGAS) and promoting pathological inflammation (Simon et al., 2019). There is ample evidence that deacetylation by SIRT6 is highly substrate specific, with only a few histone acetylation sites and non-histone substrates of SIRT6 reported. SIRT6 catalyzes robust histone deacetylation on nucleosomes (Tasselli et al., 2016). SIRT6 exhibits weaker activity on histone peptides, indicating that its efficiency depends on the physiological state of the chromatin (Gil et al., 2013). Moreover, researchers used a new method to generate specialized acetylation nucleotides on a single defined site, clearly confirming the high efficiency and selectivity of SIRT6 deacetylation of histone 3 acetylated on Lys9 (H3K9ac), H3K18ac and H3K56ac. Furthermore, SIRT6 deacetylates nuclear H3K27ac, a chromatin marker associated with transcription enhancer elements, indicating the potential function of SIRT6 in the enhancer-regulating gene expression process (Wang et al., 2016). SIRT6 deacylation activity is estimated to be at least two orders of magnitude higher than its deacetylation due to increased affinity for the fatty acid moiety (Jiang et al., 2013). SIRT6 regulates the secretion of TNF-α through hydrolysis of the long-chain fatty acid lysine, which seems highly significant for airway inflammation (Etchegaray et al., 2013).

SIRT7 is located in the nucleoli and functions in association with active ribosomal RNA (rRNA) genes. SIRT7 and rRNA interact closely with RNA polymerase I (Pol I) (Ford et al., 2006). SIRT7 is necessary for rDNA transcription, activating RNA Pol I. Recent studies have identified the role of SIRT7 in DNA repair. In response to DNA DSBs, similar to SIRT6, SIRT7 accumulates near DNA break sites, especially acetylated lysine 18 of histone H3 (H3K18ac) deacetylase, facilitating the recruitment of p53-binding protein 1 (53bp1) (Zhang et al., 2016). Focal accumulation of 53bp1 promotes nonhomologous end-joining in response to DNA damage. Apart from its deacetylase activity, the biochemical functions and the specific chromatin modifications associated with SIRT7 are still unclear. SIRT7 has been found to increase the stress resistance of cardiomyocytes, while SIRT7-deficient mice showed heart abnormalities and a shorter life expectancy (Vakhrusheva et al., 2008). Analogously, SIRT7 prevents endoplasmic reticulum (ER) stress and fatty liver disease by regulating H3K18Ac and inhibiting Myc activity in the liver (Shin et al., 2013).

## 3 Applications of SIRTs in Inflammatory Respiratory Disease

### 3.1 Regulatory Effect of SIRTs on BA

BA is one of the most common chronic inflammatory disorders. Asthma severely limits airflow in the lungs (Lefaudeux et al., 2017). This limited airflow leads to airway remodeling, and contractile agonists, growth factors, and inflammatory mediators contribute to hyperproliferation, excessive mass, and enhanced cell migration of airway smooth muscle (ASM) cells in the airway wall. To date, based on inflammatory cell counts in tissue and blood, two main subtypes of type 2 inflammation have been defined in asthma: T helper type 2 cell high (T2-high) and T helper type 2 cell low (T2-low) (Zhu et al., 2018). Increased expression of a Th2 transcription factor in the airways of asthmatic patients suggests that protein deacetylases, such as type III histone deacetylases, might regulate its function (Cosío et al., 2004). Alterations in SIRT levels in the lungs may influence the development of airway inflammation (Ma et al., 2019).

SIRT1 plays a role in maintaining the Th2 cell balance and preventing sustained cellular differentiation toward a Th2 phenotype (Colley et al., 2016). In some studies (Wang et al., 2015a; Zhang et al., 2019), serum SIRT1 levels were positively correlated with serum IgE levels and negatively correlated with pulmonary function. In addition, the interaction between IgE and antigens causes a series of immunological reactions in asthma (Oettgen and Geha, 1999). SIRT1 might play a dual role in airway inflammation, and the effects of SIRT modulators in BA may depend on the stage and severity of the disease (Ma et al., 2019). However, blocking SIRT2 and activating SIRT6 may attenuate the symptoms of BA (Lee et al., 2019). Additionally, SIRT7 is involved in the proliferation and migration of TGF-β-treated mouse ASM cells, regulating the expression of the TGF-β receptor I (TβRI) protein, which suggests that it may be involved in the regulation of airway remodeling in asthma (Fang et al., 2018).

### 3.2 Regulatory Effect of SIRTs on CRS

CRS is characterized by mucosal inflammation in the sinuses and paranasal sinuses and mucosal changes, including inflammatory thickening and nasal polyp formation. The formation of nasal polyps is related to hypoxic factors and Th2-type immune responses; that is, a hypoxic environment can affect nasal mucosal inflammation and epithelial cell remodeling and promote the formation of nasal polyps (Shun et al., 2016). Studies have shown that SIRT1 helps to inhibit nasal polyp formation. Overexpression of SIRT1 downregulates hypoxia inducible factor (HIF)-1α activity, thereby reversing hypoxia-induced epithelial-mesenchymal transformation (EMT) of lung epithelial cells and inhibiting nasal polyp formation (Chelladurai et al., 2021). In addition, nasal polyp tissues exhibit a decrease in SIRT6 expression and an increase in lactate dehydrogenase and Beclin1 expression. The overexpression of SIRT6 in primary fibroblasts extracted from nasal polyps inhibited the expression of lactate dehydrogenase (LDH), and hypoxia may promote the formation of nasal polyps by promoting autophagy of nasal polyp fibroblasts (Shun et al., 2016). In other words, regulation of glycolytic activity to promote autophagy of fibroblasts may have potential for the future treatment of nasal polyps. Furthermore, the upregulation of SIRT6 gene expression in nasal mucosal epithelial cells can inhibit the migration of high mobility group protein 1 (HMGB1) induced by lipopolysaccharide (LPS), suggesting that SIRT6 may inhibit the development of nasal polyps by altering inflammatory processes (Chen et al., 2017).

### 3.3 Regulatory Effect of SIRTs on COPD

COPD, a leading cause of mortality and morbidity, has become a major health problem worldwide. COPD affects approximately 10% of people over 45 years of age (Burney et al., 2015). Melatonin blocks the development of COPD and has been found to increase SIRT1 expression in lung tissue in mice with COPD. These findings were attributed to attenuation of airway inflammation via SIRT1-dependent inhibition of the NLRP3 inflammasome and IL-1β in rats with COPD (Peng et al., 2018).

Oxidative and carbonyl stress occur in the lungs of patients with chronic obstructive pulmonary disease and smokers, as well as in rodents exposed to cigarette smoke (CS) (Kirkham and Barnes, 2013). However, the anti-aging protein SIRT1 enhances resistance to oxidative stress (Alcendor et al., 2007). Due to its importance in regulating the oxidative stress response, SIRT1 dysfunction is associated with age-related diseases such as COPD (Russomanno et al., 2017). Studies have shown that FOXO3 knockout mice show an increase in lipid peroxidation products and a decrease in antioxidant genes and enzymes, which cannot be weakened by a SIRT1 activator (SRT1720). This suggests that FOXO3 is involved in SIRT1-mediated CS-induced oxidative stress regulation. Thus, SIRT1 prevents pulmonary oxidative stress caused by cigarette smoke through a FOXO3-dependent mechanism (Yao et al., 2014). Moreover, matrix melloproteinase-9 (MMP9) is involved in the decomposition and aging of the extracellular matrix during disease progression, and the expression of macrophages in COPD patients increases as the disease progresses. Moreover, CS also led to a decrease in SIRT1 expression in mouse lung tissues when MMP9 was elevated. One study showed that MMP9 expression in the lungs decreased after intranasal use of SRT2172. Therefore, SIRT1 is a negative regulator of MMP9 expression (Nakamaru et al., 2009).

Studies have revealed severe inflammation in the airway of COPD mouse models, along with alveolar space enlargement and mitochondrial damage. SIRT3 may inhibit mitochondrial oxidative stress in the airway epithelium by regulating MnSOD, thereby promoting the pathogenesis of COPD (Zhang et al., 2020). SIRT4 expression is significantly downregulated in CS-treated human pulmonary microvascular endothelial cells (HPMECs). Overexpression of SIRT4 significantly inhibits CS-induced adherence of monocytes to HPMECs. In addition, CS-induced NF-KB activation is negatively regulated by inhibiting the degradation of IκBα. Therefore, SIRT4 protects HPMECs exposed to CS through a mechanism that may involve the NF-KB pathway (Chen et al., 2014b). Moreover, CS-induced SIRT5 deacetylizes FOXO3 and prevents apoptosis of lung epithelial cells, thus protecting against COPD (Wang et al., 2015b).

### 3.4 Regulatory Effects of SIRTs on ALI and ARDS

ALI and ARDS are characterized by the activation of macrophages and neutrophils and the disruption or edema of the alveolar capillary membrane barrier, all of which can lead to respiratory failure (Thompson et al., 2017). One study implicated SIRT3 mitochondrial superoxide formation and macrophage reprogramming as key mediators for increased inflammation and severity of lung damage in animal models of endotoxin-induced lung damage (Kurundkar et al., 2019). In addition, SIRT1 is important for mitochondrial metabolism, energy homeostasis and oxidative stress because it regulates metabolic pathways, including the TCA cycle and oxidative phosphorylation (OXPHOS) (Kitada et al., 2013). Mitochondrial stress in SIRT1-expressing mouse bone marrow-derived dendritic cells (BMDCs) was evident due to reduced basal and maximal respiration rates, leading to decreased ATP production. Surprisingly, at low concentrations, ROS regulate homeostatic signaling cascades, while at higher concentrations, they oxidize DNA, lipids, and crucial signaling proteins, leading to cell damage and inflammation (Garofalo et al., 2013). In one study, p65 phosphorylation in the lung tissue of ARDS mice was inhibited by a SIRT1 activator. SIRT1 increases the inflammatory response and oxidative stress in LPS-induced ARDS (Zhan et al., 2021). A recent study found that SIRT6 promotes macrophage M2 polarization to relieve ARDS caused by sepsis in an autophagic-dependent and nonautophagic-dependent manner (Wang et al., 2022).

**FIGURE 4:**
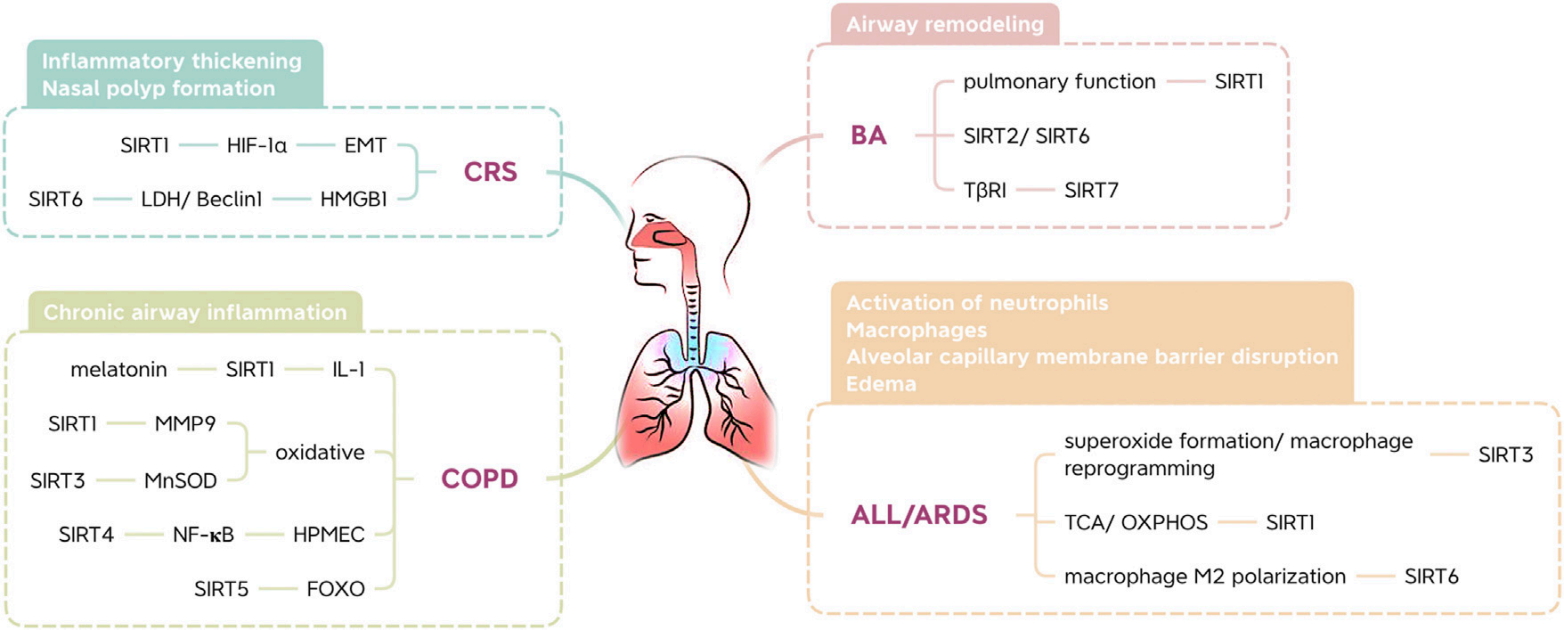
The applications of SIRTs in inflammatory respiratory disease.

Given the critical role of SIRTs in a variety of human respiratory diseases, including BA, CRS, COPD, ALI, and ARDS, the use of small-molecule modulators to modulate the activity of SIRTs may provide therapeutic benefits for blocking the initiation and progression of these diseases (Figure 4).

## 4 SIRT Modulators

### 4.1 Recent Laboratory Studies on SIRT Modulators

**FIGURE 5:**
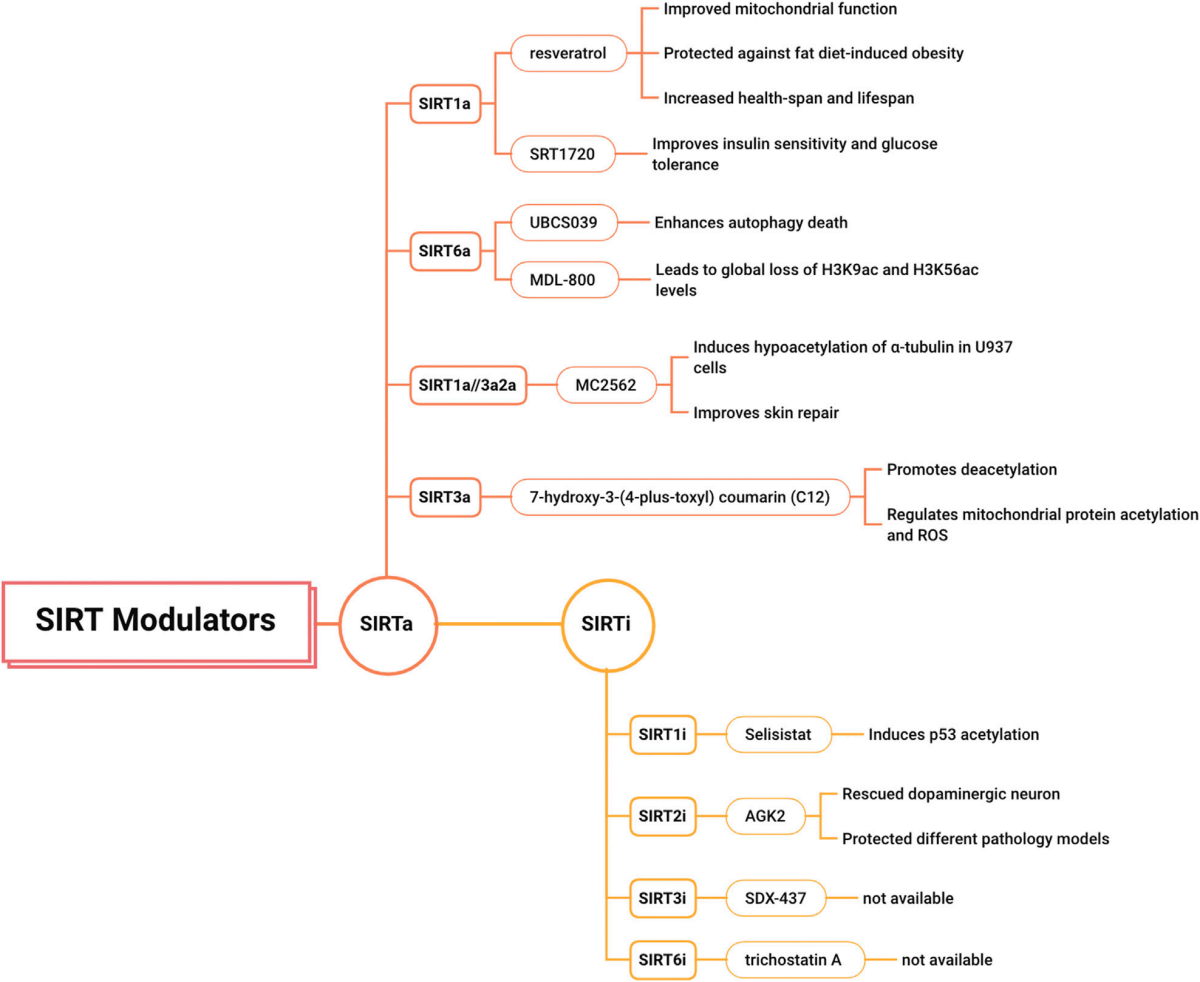
The modulators of SIRTs.

Although understanding of this family of broad-spectrum protein lysine deacylases has increased dramatically in recent years, many questions remain. The search for heterospecific modulators (including SIRT activators (SIRTas) and inhibitors (SIRTis)) is still in its infancy. Therefore, many relevant SIRT modulators have been described in the literature and in patents. For example, dietary SRT1720 (SIRT1a) improves insulin sensitivity and glucose tolerance in genetically obese mice, leading to beneficial effects on lipid metabolism and weight loss (Milne et al., 2007). UBCS039 (SIRT6a) enhances autophagic death in various cancer cell lines by activating the dependency mechanism through SIRT6 (Iachettini et al., 2018). MDL-800 (SIRT6a) leads to global reduction of H3K9ac and H3K56ac levels in human hepatocellular carcinoma cells (HCCs), as demonstrated in tumor xenograft models (Zhimin Huang et al., 2018). MC2562 (SIRT1a/2a/3a) induces hypoacetylation of α-tubulin in U937 cells and is not released from HaCaT cells. In addition, MC2562 improves skin repair in mouse wound healing models and exhibits antiproliferative effects by reducing H4K16 acetylation in various cancer cell line panels (Valente et al., 2016). One recent study identified a new type of SIRT3 activator, 7-hydroxy-3-(4-plus-toxyl) coumarin (C12), which has a high affinity for SIRT3, promotes deacetylation of MnSOD, and regulates mitochondrial protein acetylation and ROS (Rangarajan et al., 2015). Selisistat (SIRT1i) induces p53 acetylation in different cancer cell lines; however, it does not affect cell proliferation or viability (Süssmuth et al., 2015). AGK2 (SIRT2i) rescued dopaminergic neurons exposed to α-synuclein toxicity and protected different pathology models against developing Parkinson’s disease (PD) (Outeiro et al., 2007). SDX-437 (SIRT3i) and trichostatin A (SIRT6i) are not currently available for clinical use (You and Steegborn, 2018) (Figure 5).

### 4.2 Clinical Trials Evaluating SIRT Modulators for Respiratory Conditions or Inflammation

The lack of well-described pharmacological characteristics of these small-molecule compounds has hindered their translation from the laboratory to clinical use (Dai et al., 2018). In the case of activators, binding sites are usually not easily detected by crystal structure examination, and there is no general and systematic strategy to identify and target these sites (Anderson et al., 2017).

SIRT1 is the most studied isotype to date, followed by SIRT2 and SIRT3. Thus, a few small-molecule modulators targeting the other isotopes have been developed, and most of the regulators evaluated in clinical trials target SIRT1. Resveratrol (RSV) is a mediator of the cardioprotective effects of wine, sparking interest in RSV as a potential therapeutic agent (Bertelli et al., 1995). This compound activates SIRT1 deacetylase activity by reducing the KM of NAD+ and acetylated peptides. Therefore, it is debatable whether the observed therapeutic effect of RSV is due to SIRT1 activation. In human trials, RSV absorption rates of approximately 70% were observed, but unmodified RSV could not be detected 30 min after administration (Walle et al., 2004). Most trials in which RSV had a positive effect employed higher doses, usually more than 500 mg/day. Whether RSV directly activates SIRTs when administered orally is unknown, but it is an easily accessible natural product with few adverse reactions. Despite showing early promise in the laboratory, the current status of RSV suggests that the development of better, more selective SIRT activators is needed. Researchers have found SIRT1 modulators structurally different from RSV that can enhance SIRT1 levels (Milne et al., 2007). Additionally, HTS was performed on a library of 280,000 compounds using fluorometry, and indoles were identified as viable scaffolds for SIRT1 targeting (Napper et al., 2005).

**FIGURE 6:**
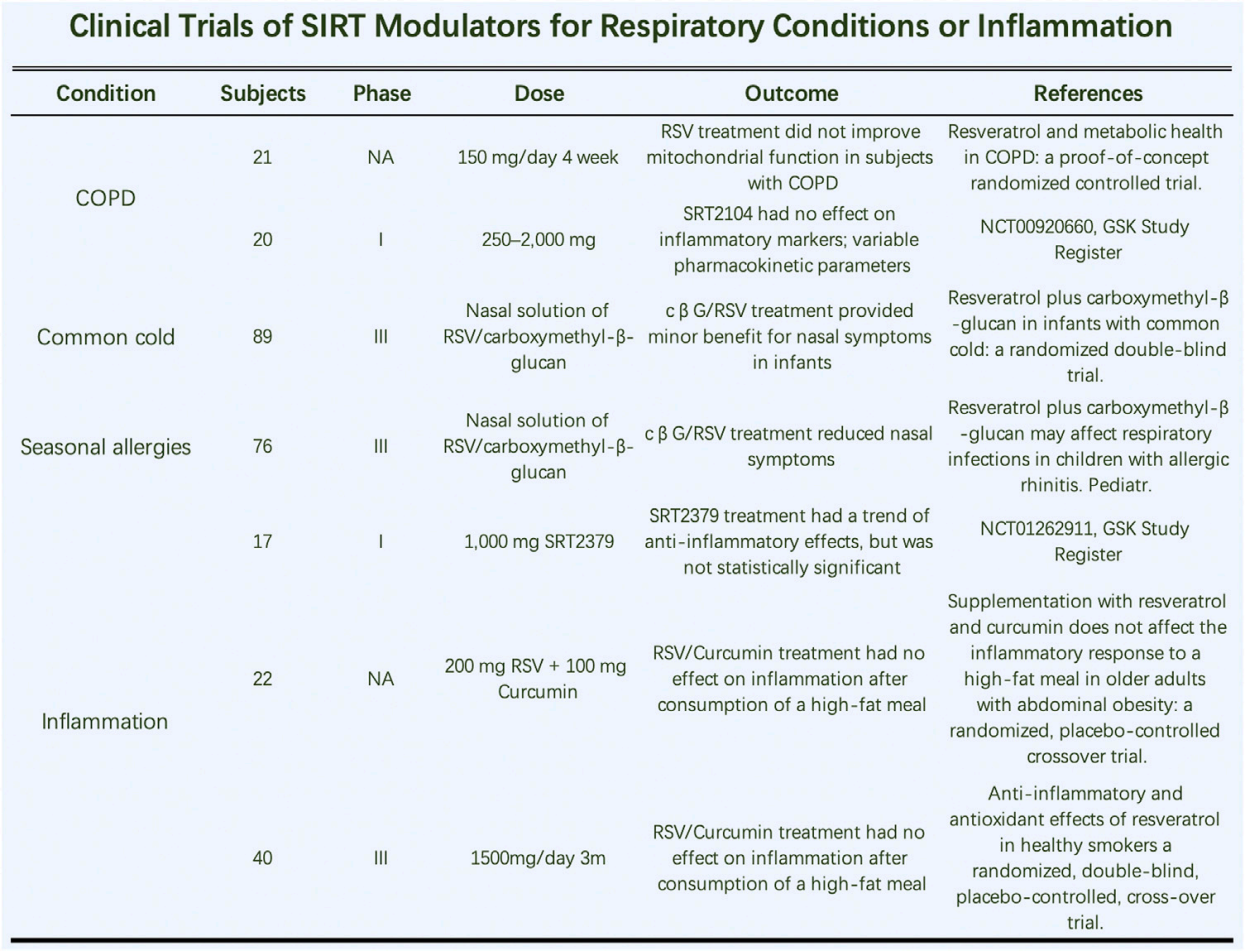
Clinical trials of SIRT modulators for respiratory conditions or inflammation.

Further study needs to be done on the application of SIRT modulators in inflammatory diseases of the respiratory system (Figure 6). RSV treatment did not improve mitochondrial function in COPD patients (Beijers et al., 2020). However, one study showed that it inhibited M1-type macrophages by SOCS3 signaling in LPS-induced ALI. Therefore, modulating macrophage subtypes by targeting SOCS3 signaling is a promising treatment for future treatments in patients with ALI and ARDS (Hu et al., 2019). Additionally, SRT2104 (SIRT1a) had no effect on inflammatory markers but influenced various pharmacokinetic parameters. Two phase III clinical trials indicated that a nasal solution of RSV/carboxymethyl-β-glucan had little effect on nasal common cold symptoms in infants (Baldassarre et al., 2020). In clinical studies of inflammatory markers in smokers, RSV treatment had beneficial effects on specific inflammatory markers and antioxidant levels. The anti-inflammatory and antioxidant effects of RSV were also evident in healthy smokers in a randomized, double-blind, placebo-controlled, crossover trial. In the same year, similar articles showed that RSV treatment had beneficial effects on several inflammatory markers and reduced fasting insulin levels. Thus, RSV may be used for the primary prevention of atherosclerosis; clinical trial evidence indicates it improves gene expression in the vascular endothelium.

## 5 Conclusion

This review provides an up-to-date overview of the most relevant biometrics of SIRT enzymes and SIRT formulations reported in the scientific literature. These small-molecule modulators are inhibitory compounds, and their selectivity regarding other SIRTs has not yet been fully evaluated. Future rational inhibitor design and direct high-throughput screening of all SIRTs, especially mammalian homologs, will undoubtedly lead to the development of highly selective and powerful inhibitors that will provide the necessary tools for elucidating the cellular function of these enzymes and may lead to the development of treatments targeted toward individual SIRTs. SIRT-targeting drugs are promising therapeutics, and advances in the respiratory field will accelerate the development of small-molecule drug candidates. If additional SIRT modulators are translated to the clinic, a surprising amount of progress will be made in targeted precision therapy for otolaryngology and pneumology.

## Abbreviations

53bp1, p53-binding protein 1; AceCS2, acetyl coenzyme A synthetase 2; Agt5, angiotensinogen 5; Agt7, angiotensinogen 7; ALI, acute lung injury; ARDS, acute respiratory distress syndrome; ASM, airway smooth muscle; BA, bronchial asthma; BMDCs, bone marrow-derived dendritic cells; cDNA, complementary DNA; cGAS, cyclic GMP–AMP synthase; COPD, chronic obstructive pulmonary disease; CRS: chronic sinusitis; DSB, double-strand break; EMT, epithelial-mesenchymal transformation; ER, endoplasmic reticulum; FOXO, forkhead box O; GDH, glutamate dehydrogenase; H3K9ac, histone 3 acetylated on Lys9; HAT, histone acetyltransferase; HDACs, histone deacetylases; HIF, hypoxia inducible factor; HMGB1, high mobility group protein 1; JNK2, C-Jun N-terminal kinase 2; L1, LINE1; LDH, lactate dehydrogenase; LPS, lipopolysaccharide; MEFs, mouse embryonic fibroblasts; MnSOD, manganese-dependent superoxide dismutase; mROS: mitochondrial ROS; NAD, nicotinamide-adenine dinucleotide; NF-KB, nuclear factor KB; OXPHOS, oxidative phosphorylation; PARP1, poly (ADP-ribose) polymerase 1; PD, Parkinson's disease; PDH, pyruvate dehydrogenase complex; Pol I, polymerase I; ROS, reactive oxygen species; rRNA, ribosomal RNA; RSV, resveratrol; SB, sodium benzoate; Sir2, silent information regulator 2; SIRTas, SIRT activators; SIRTis, SIRT inhibitors; SIRTs, sirtuins; TCA, tricarboxylic acid; TGF-β1, transforming growth factor-β1; Th2, T helper 2 cells; TβRI, TGF-β receptor I.
